# Transmission studies of chronic wasting disease to transgenic mice overexpressing human prion protein using the RT-QuIC assay

**DOI:** 10.1186/s13567-019-0626-2

**Published:** 2019-01-22

**Authors:** Brent Race, Katie Williams, Bruce Chesebro

**Affiliations:** Laboratory of Persistent Viral Diseases, Rocky Mountain Laboratories, National Institute of Allergy and Infectious Diseases, National Institutes of Health, 903 South Fourth Street, Hamilton, MT 59840 USA

## Abstract

Chronic wasting disease (CWD) is a fatal prion disease which infects deer, elk and moose. CWD was first described as a wasting syndrome in captive deer in Colorado and Wyoming wildlife facilities from 1967 to 1979. Currently, CWD has been reported in 26 states of the USA, three Canadian provinces, South Korea, Norway and Finland. Since human consumption of cervids is common, it is critical to determine if CWD can infect humans. Published research, including epidemiologic studies and transmission studies using animal models, including transgenic mice that express human prion protein, have suggested existence of a strong species barrier between cervid CWD and humans. In the current study, we tested CWD transmission into two additional strains of transgenic mice (tg66 and tgRM). These mice over-express human prion protein at high levels and are highly sensitive to infection by human-tropic prions. One hundred and eight mice were inoculated intracerebrally with three different sources of CWD. After long periods of observation, brain tissues from CWD-inoculated mice were screened for evidence of prion infection by RT-QuIC, immunohistochemistry (IHC) and immunoblot. No IHC or immunoblot evidence was found to suggest transmission had occurred, and most mice were negative by RT-QuIC assay. However, four mice with inconsistent positive RT-QuIC reactions were detected. The seeding activity detected in these mice may represent a low level of CWD agent, suggesting a possible transfer of CWD infection. Alternatively, these results might be due to false positive reactions or residual CWD inoculum.

**Electronic supplementary material:**

The online version of this article (10.1186/s13567-019-0626-2) contains supplementary material, which is available to authorized users.

## Introduction

Chronic wasting disease (CWD) is a transmissible spongiform encephalopathy (TSE) or prion disease of deer, elk, moose and reindeer. CWD was first described as a wasting syndrome in captive deer held in wildlife facilities in Colorado and Wyoming [1]. Human transportation of CWD-infected cervids to new areas and natural spread of CWD from infected populations to surrounding areas have both contributed to the spread of CWD in North America [2–4]. CWD has now been detected in 26 states of the USA, Canada, South Korea, Norway and Finland. The combination of long-term environmental stability, relative ease of transmission, and the lack of an effective treatment or vaccine against CWD makes it very likely that CWD will continue to spread [2–4]. As the range and prevalence of CWD increases, so does the potential for human exposure to CWD prions. Assessing the risk CWD poses toward human health is critical toward protecting consumers of cervids and cervid-derived products.

Transmission of prion diseases from animals to humans have shown variable outcomes. One of the longest recognized animal prion diseases, sheep scrapie, has been present for over 200 years. However, despite human exposures to scrapie, there is minimal evidence that transmission of scrapie to humans has ever occurred [5]. In contrast, there is strong evidence of transmission of bovine spongiform encephalopathy from cattle to humans, albeit at very low frequency [6–8]. It is currently unknown whether CWD can be transmitted from cervids to humans.

Epidemiology and case studies have shown no conclusive link of CWD exposure to an increase in human prion disease [9]. However, these data could miss subclinical infections and infrequent cross-species transmission events. Scientists have also assessed the likelihood of CWD transmission from cervids to humans using laboratory models [9–11]. Collectively, results from several in vitro experiments suggest that a strong species barrier exists between cervids and humans [9]. In vivo experimental transmission studies using two species of non-human primates (NHP) gave differing results. Squirrel monkeys were susceptible to CWD by intracerebral and oral infection [12–14], but no cynomolgus macaques showed evidence of infection by clinical evaluation or laboratory testing [15]. In previous studies, cynomolgus macaques and humans appear to have similar patterns of susceptibility to known human prion diseases, while squirrel monkeys appear to be a more permissive host to many strains of prion agents. From an evolutionary standpoint, macaques are a closer genetic match to humans than squirrel monkeys. However, when comparing only the prion protein gene sequence, both monkey species differ from humans and from each other [13, 16]. Thus, these genetic data do not provide a simple explanation of different CWD transmission results in these two non-human primate species.

Experiments using transgenic mice expressing non-mouse PrP have shown that PrP itself appears to be the most important factor in determining the barrier to cross-species prion infection [11, 17–19]. Transgenic mice that express human prion protein (humanized) have been shown to be susceptible to a variety of human-derived prions and have been used as a surrogate test for the susceptibility of humans to CWD infection by several groups [20–24]. Collectively, the humanized mice tested in these studies varied in prion protein expression levels from 1 to 6× physiologic levels and the amino acid residue at position 129 (both M and V have been tested) [9]. Additionally, the CWD used for each study appears to be from multiple sources. Screening for CWD transmission to recipient mice in all the studies included observations for clinical signs, IHC staining for PrPSc, and analysis for neuropathology consistent with prion disease. In each study, except for Wilson et al. [21], brains were also screened by immunoblot assay for protease resistant PrP. Wilson et al. [21] used an antigen capture immunoassay (Idexx herdChek) as an additional screening tool. Two of the studies reported observation of clinical signs in a small number of mice, but prion disease could not be detected in these studies [21, 24], or in any of the other three published reports using humanized mice.

In our current studies we inoculated CWD intracerebrally into two different transgenic mice that overexpress human prion protein (M129) at 8–16-fold (tg66) and two to fourfold (tgRM) normal physiologic levels. Tg66 mice express extremely high levels of human prion protein and have been shown to be a very sensitive model for prion transmission [25, 26]. Following extended observation periods, brains from CWD-inoculated mice were tested for prion disease using the highly sensitive RT-QuIC assay, IHC and immunoblot. We found no IHC or immunoblot evidence for cross-species transmission of CWD to either of the transgenic lines studied, and 84 out of 88 mice screened were also negative by RT-QuIC assay. However, four mice with low levels of RT-QuIC PrP amyloid seeding activity were identified.

## Materials and methods

### Experimental mice

Generation of tg66 and tgRM transgenic mice expressing human PrP were described previously [13]. Tg66 mice were made by Richard Rubenstein and provided to RML by Robert Rohwer. Tg66 mice are on an FVB/N genetic background and are homozygous for a transgene that encodes human prion protein M129. Tg66 mice overexpress human PrP at 8–16-fold levels higher than normal physiologic levels and have been shown to be susceptible to vCJD, sCJD and mouse-adapted 22L scrapie [13, 26]. Tg66 mice do not express any normal mouse PrPsen. TgRM mice were obtained from Larissa Cervenakova of the American Red Cross. At Rocky Mountain Laboratories, tgRM mice were backcrossed to a C57 background by Suzette Priola for 10 generations. Hemizygous tgRM mice that expressed human PrPsen-M129 at a level two to fourfold higher than normal physiologic levels were used in the experiments. TgRM mice did not express any normal mouse PrPsen.

### Inocula, inoculations and clinical observations

Three pools of CWD-infected brain homogenates (MD-1, WTD-1 and Elk-2) from three different cervid species collected from different geographical regions were used. All brain tissues used for each pool were collected from animals showing clinical signs of CWD and confirmed to be CWD-positive following necropsy. MD-1 contained brains from six mule deer from Wyoming, WTD-1 contained brains from seven captive white-tailed deer from Wyoming and Colorado and Elk-2 contained brains six elk from a South Dakota game farm. Electrophoretic mobilities and glycoform ratios appeared to be very similar between the three CWD pools [13]. The level of infectivity present in each pool had been previously determined by bioassay with mice that express mule deer prion protein [13]. Based on these results the following LD_50_ were inoculated per injection: MD-1, 1.5 × 10^5^; WTD-1, 1.2 × 10^5^; Elk-2, 6.0 × 10^4^. Groups of 13–20 mice from each mouse strain (tg66 and tgRM) were inoculated with each of the CWD-infected brain homogenate pools (Table 1). Each mouse was inoculated intracerebrally with 30 µL of a 1% brain homogenate using a 27-gauge needle.

**Table 1:** **Tg66 and tgRM mice inoculated with CWD**

Mouse strain	Inoculum	Number of mice^a^	Number of mice excluded^b^	Days post-inoculation until euthanasia (range, group average)	Clinical suspects^c^
Tg66	Elk-2	17	2	600–719, 660	7/17
Tg66	MD-1	19	0	467–719, 631	2/19
Tg66	WTD-1	16	1	503–744, 685	6/16
TgRM	Elk-2	19	1	538–748, 706	1/19
TgRM	MD-1	15	5	551–748, 692	0/15
TgRM	WTD-1	11	2	496–798, 696	2/11

^a^Includes the total number of mice inoculated and observed for > 450 days.

^b^Indicates the number of mice that were inoculated but euthanized prior to 450 days and have been excluded from PrP screening tests with the exception of three tgRM mice that were euthanized at 369, 371 and 432 dpi and tested by RT-QuIC.

^c^Clinical suspects in the tg66 mice included mice that displayed one or more of the following signs: gait abnormalities, hind limb weakness, tremors, kyphosis or weight loss. Clinical suspects in the tgRM mice included two mice with abnormal gait and one thin mouse.

All mice were observed once daily by animal care staff and 1–2 times per week by prion investigators for assessment of overall health and observation for neurologic signs consistent with prion infection. In many cases, mice were observed for 450–798 days post-inoculation (dpi) and differentiation of age-related concurrent conditions from prion specific clinical signs was difficult. Mice were euthanized when they developed signs of neurologic disease, weight loss, or developed significant non-TSE diseases that negatively affected their well-being (e.g. cancer, dermatitis, respiratory difficulty). Mice euthanized prior to 450 dpi that did not have neurologic signs or weight loss were excluded from the prion disease screening assays. Tg66 mice had a higher incidence of euthanasia due to neurologic disease compared to TgRM mice (Table 1). However, neuropathology and PrPres deposition consistent with end-stage prion infection could not be confirmed in these tg66 mice and the observed signs were likely caused by other conditions. Following euthanasia, brains were removed, cut in half along the midline and processed for either histology (immersion in neutral buffered formalin) or frozen for later use in diagnostic assays.

### RT-QuIC

RT-QuIC reactions were performed as previously described using either recombinant hamster 90–231 (Ha rPrP) (Accession No. KO2234) or bank vole prion protein (BV rPrP) (residues 23 to 230; Methionine at residue 109; Accession No. AF367624) as substrates [27]. Briefly, sample brains were homogenized to 10% (w/v) in PBS. Homogenate supernatants were then collected following a 1-min clearance step at 2000 × *g*. Samples were then tenfold serially diluted in either 0.1% SDS (sodium dodecyl sulfate, Sigma)/PBS/N2 (Gibco) for Ha substrate or 0.05% SDS/PBS/N2 for BV substrate to yield 10^−3^ brain tissue concentrations. 2 µL sample volumes were added to reaction wells of a black 96-well, clear bottom plate (Nunc) containing 98 µL of RT-QuIC reaction mix, resulting in final concentrations of 0.002% SDS for Ha reactions or 0.001% SDS for BV substrate reactions, 10 mM phosphate buffer (pH 7.4), 300 mM NaCl, 0.1 mg/mL rPrPsen substrate, 10 μM thioflavin T (ThT), 1 mM ethylenediaminetetraacetic acid tetrasodium salt (EDTA). The plate was then sealed with a plate sealer film (Nunc) and incubated at 50 °C for Ha substrate or 42 °C for BV substrate, in a BMG FLUOstar Omega plate reader with a repeating protocol of 1 min shaking (700 rpm double orbital) and 1 min rest throughout the indicated incubation time. ThT fluorescence measurements (450 ± 10 nm excitation and 480 ± 10 nm emission; plate bottom read) were taken every 45 min.

The plate reader gain was consistent for each run, as were the concentrations of thioflavin T and SDS in each reaction. The maximum fluorescence readout on our plate reader is 260 000 units. For all runs gain was set at 1600, and this setting resulted in maximum fluorescence values averaging 75 000 for wells from a known sCJD-infected tgRM mouse positive-control brain sample, and values of 4000 to 6000 for negative-control uninfected brain samples. Four replicate wells from the same positive control mouse were run on each tray (tgRM sCJD #F461 at a 10^−3^ dilution). The highest florescence level measured for this control mouse was defined as the 100% normalized florescence level for that specific tray. Data from each well were then normalized by dividing the fluorescent readings of each well by the florescence level equaling 100%.

**Figure 1:**
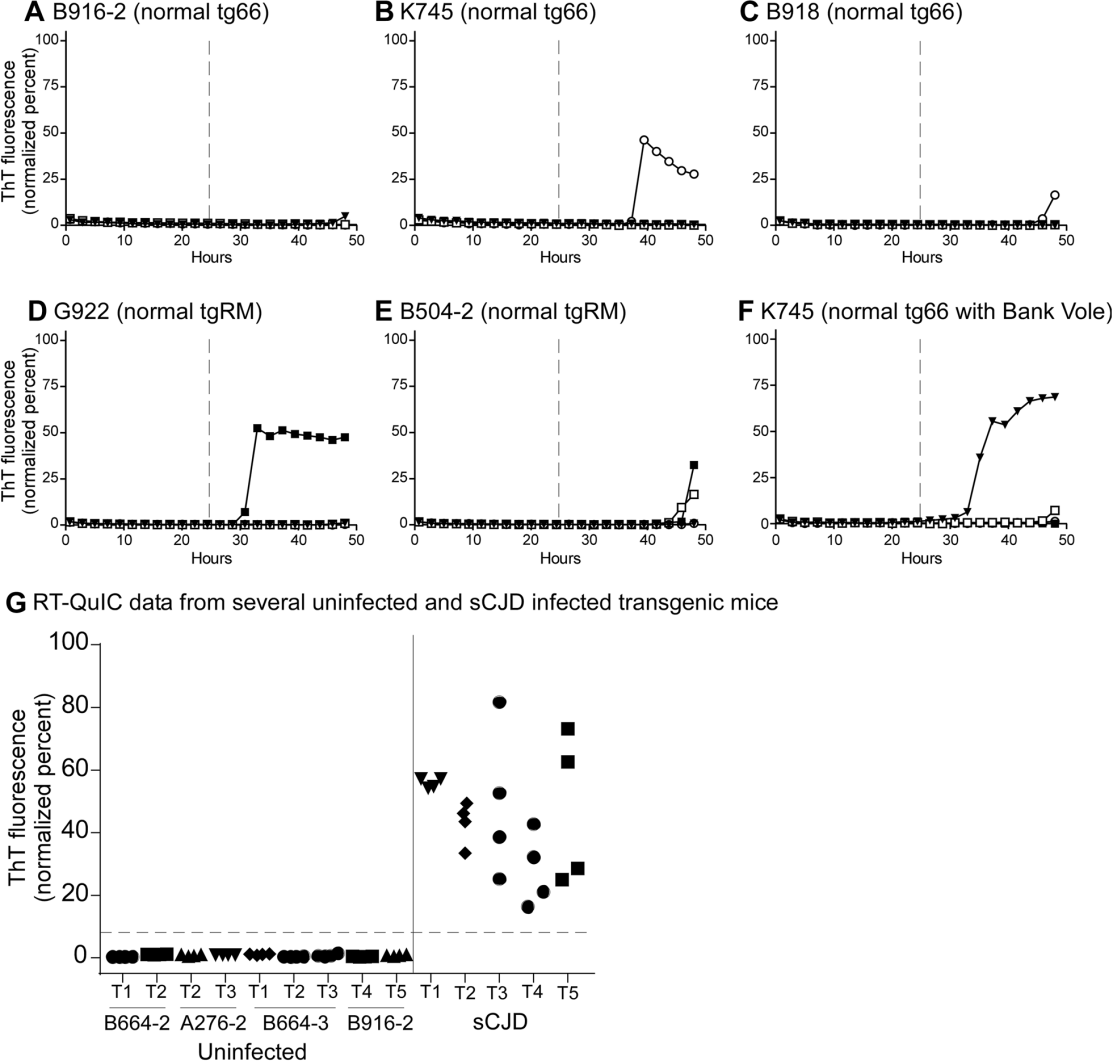
**RT-QuIC analysis of uninoculated and sCJD inoculated transgenic mice.** All brain samples were tested at a 10^−3^ dilution. In **A**–**F**, four independent wells from each mouse are represented as individual curves with unique symbols. **A**–**E** Data from five different uninoculated tg66 and tgRM mice tested with Ha rPrP substrate. **F** An example of an uninoculated tg66 mouse tested with BV rPrP substrate. **G** Ha rPrP RT-QuIC data from several replicate trays of uninoculated transgenic mice (left side) and sCJD-positive control (right side) transgenic mice at the 25-h timepoint. Each point represents measured fluorescence in a single test well. Different tray numbers and different mice are indicated below the x-axis. A horizontal dashed line is shown at 10% florescence (27 standard deviations above baseline florescence) that clearly separated negative and positive reactions.

In addition, at least four negative control wells were run at a 10^−3^ on each tray, with most trays having an average of 12 negative control wells representing three uninfected control mice. Negative controls included brain samples from nine uninoculated tg66 mice, 5 tg66 mice inoculated with normal tg66 brain and four uninoculated tgRM mice. In previous reports using RT-QuIC under various conditions, spontaneous appearance of ThT fluorescence has been reported in negative control samples after prolonged reactions times. Therefore, to identify valid positive fluorescence signals, it was important to determine empirically the latest reaction times which did not have any false-positive fluorescence. In the conditions used here with both hamster and bank vole rPrP, such spontaneous fluorescence in negative samples was never noted prior to 25 h reaction time. For hamster rPrP, spontaneous fluorescence was rare (3.5%) between 25 and 50 h with the earliest detection at 31 h (Figures 1B–E). For bank vole rPrP, spontaneous fluorescence signals in negative controls were more common (30%), but again always occurred later than 25 h (Figure 1F).

In contrast, at the 25 h reaction timepoint, positive control samples had normalized fluorescence values ranging from 18 to 80%, and as seen in Figure 1G, there was a significant gap between the negative control values (mean 0.7%) and the lowest positive control values (18%). Therefore, we assumed a cutoff value of 10% at the 25 h timepoint to separate positive from negative values. Based on the mean and SD of negative control values from 36 different wells at 25 h (Figure 1G) (0.7 ± 0.35), the 10% cutoff value would be 27 standard deviations above the mean for the negative controls.

For the initial screening of each sample, four independent wells were tested at a 10^−3^ dilution for each mouse brain. In most cases, the values of replicate wells were closely clustered at low values, similar to the negative control samples. In some cases, positive wells were observed in experimental mice and additional assay trays were run using from 4 to 16 wells for each experimental animal. Increasing the well number was found to be the best way to obtain more statistical power in these analyses (Table 2). Experimental samples were compared to negative control wells on the same tray using Fischer’s exact test for 2 × 2 contingency tables.

**Table 2:** **Summary of RT-QuIC results for suspect positive tg66 mice inoculated with CWD**

Mouse #	Inocula	dpi	RT-QuIC recombinant PrP substrate	
			Bank vole^a^	Hamster 90–231^a^
B351-3	Elk-2	662	0/4, 0/4	1/4, 1/4, 2/4
B349-1	Elk-2	651	0/4, 0/4	2/4, 0/4, 1/4, 4/12
B377-4	WTD-1	710	0/4, 0/4	3/4, 1/4, 1/4, 9/12
B378-3	WTD-1	717	2/4, 1/4, 3/4	8/8, 0/4, 0/4, 11/16

^a^Each independent RT-QuIC assay is shown as a separate fraction where the numerator indicates the number of positive wells over the total number of wells tested (denominator) in that specific run.

### Immunohistochemistry (IHC)

Tissues were removed and placed in 10% neutral buffered formalin for 3 to 5 days. Following fixation, brains were processed by dehydration and embedding in paraffin. Sections were cut using a standard Leica microtome, placed on positively charged glass slides, and air-dried overnight at room temperature. On the following day slides were heated in an oven at 60 °C for 20 min. Neuropathology was assessed on hematoxylin and eosin (H&E) stained sections. H&E staining was performed according to the manufacturer’s (Shandon) instructions; hematoxylin incubation of 12 min, eosin incubation of 4 min.

All IHC, deparaffinization, antigen retrieval and staining were performed on the automated Discovery XT staining system (Ventana Medical Systems). “No-primary” antibody controls were run on a sub-set of slides for each antibody detection system.

For staining of prion protein using biotinylated monoclonal anti-prion antibody 3F4 (b3F4) (Covance Research Products), antigen retrieval was done using a Biocare Medical DC2002 decloaking chamber and citrate buffer pH 6.0 for 20 min at 120 °C and 20 PSI. Discovery S Block RUO (Ventana 760-4212) was applied for 4 min to block non-specific binding of the primary antibodies. Primary antibody b3F4 was applied for 60 min at 37 °C. PrP staining was completed using a DABMap chromogen detection kit that contains SA-HRP (Ventana 760-124). Hematoxylin was applied as a counterstain. For staining of prion protein using anti-prion rabbit polyclonal antibody EP1802Y (GeneTex #GTX61655), antigen retrieval was done using the Discovery XT system with the extended CC1 protocol (cell conditioning buffer containing Tris–Borate-EDTA, pH 8.0, incubated 44 min at 100 °C. Discovery S Block RUO (Ventana 760-4212) was applied for 4 min to block non-specific binding of primary and secondary antibodies. Primary antibody EP1802Y was applied at a 1:18 000 dilution for 60 min at 37 °C. The secondary antibody was biotinylated goat anti-rabbit IgG (Biogenex Ready-to-use Super Sensitive Rabbit Link) applied for 32 min at 37 °C. For the anti-PrP staining, staining was completed using a DABMap chromogen detection kit and hematoxylin counterstain. Both PrP antigen retrieval steps described above do not destroy normal cellular, non-disease associated PrP (PrPsen), and a background level of PrPsen staining can be seen in tg66 mice.

For GFAP staining antigen retrieval was done using the Discovery XT system with the mild CC1 protocol (cell conditioning buffer containing Tris–Borate-EDTA, pH 8.0, incubated 12 min at 100 °C). The anti-GFAP antibody was used at a dilution of 1:3500 in antibody dilution buffer, applied for 16 min at 37 °C. The secondary antibody was biotinylated goat anti-rabbit IgG described above and was applied for 16 min at 37 °C. Staining was completed using a RedMap detection kit and hematoxylin counterstain.

Sections stained with H&E, b3F4, EP19802Y and GFAP were scanned with an Aperio ScanScope XT (Aperio Technologies, Inc.) and analyzed and photographed using Aperio Imagescope software.

### Immunoblotting for detection of PrP

Brain tissue was homogenized in 1× PBS as a 20% (wt/vol) tissue homogenate using a mini-bead beater for 45 s on the homogenization setting. Aliquots were stored at −20 °C. For routine detection of proteinase K-resistant PrP (PrPres), samples were treated with proteinase K (PK) at 50 μg/mL. Briefly, 20 μL of a 20% homogenate from each sample was adjusted to 100 mM Tris–HCl (pH 8.3), 1% Triton X-100, 1% sodium deoxycholate and 50 μg/mL PK in a total volume of 31 μL. Samples were incubated for 45 min at 37 °C. All PK digestions were stopped by adding 2 μL of 100 mM Pefabloc (Roche Diagnostics), and the reaction mixture was placed on ice for 5 min. An equal volume of 2× Laemmli sample buffer (Bio-Rad, Hercules, CA, USA) was added and the samples boiled for 5 min. Samples were frozen at −20 °C until needed for gel electrophoresis (see electrophoresis description in the paragraph below).

For the sodium phosphotungstic acid (PTA) precipitation of PrPres we followed a previously published procedure [28] with slight modifications. Eleven CWD-inoculated and two uninoculated, age-matched controls were analyzed. Homogenates (described above) were diluted in PBS to create 10% homogenates. 500 µL of a 10% BH was mixed with an equal volume of 4% Sarkosyl, vortexed, and incubated in a water bath at 37 °C for 30 min. Benzonase (5 U/µL) and magnesium chloride (0.2 M) were then added to final concentrations of 25 U/mL and 0.001 M, respectively. Samples were vortexed and incubated in a water bath at 37 °C for 45 min. Centrifugation at 5000 × *g* for 5 min at room temperature was performed, and the supernatant was transferred to a new tube. PK was added to a final concentration of 50 µg/mL, and the mixture was vortexed and incubated in a water bath at 37 °C for 1 h. The reaction was stopped with a 5 mM final concentration of Pefabloc. Four percent sodium PTA and 34 mM magnesium chloride, pH 7.4, were added to final concentrations of 0.3% and 2.73 mM, respectively, and the solution was incubated in a water bath at 37 °C for 1.5 h. Samples were then centrifuged at 16 000 × *g* for 30 min at 37 °C, and the supernatants were discarded. Pellets were then resuspended in 200 µL of PBS-EDTA (40 mL of 0.5 M EDTA and 60 mL of PBS, pH 7.4), incubated for 30 min in a 37 °C water bath, and then centrifuged at 16 000 × *g* for 30 min at 37 °C. The supernatants were again discarded, and the pellet was resuspended in 44 µL of Laemmli sample buffer, vortexed, and boiled for 5 min. 20 µL was loaded into a single lane on a 16% Tris–glycine gel (Invitrogen, Thermo Fischer Scientific).

Both PTA precipitated and non-PTA treated samples were electrophoresed on a 16% Tris–glycine sodium dodecyl sulfate–polyacrylamide gel electrophoresis (SDS-PAGE) gel (Life Technologies, CA, USA) and blotted to polyvinylidene difluoride (PVDF) membranes using a 7-min transfer on an iBlot (Life Technologies) device. Immunoblots were probed with the anti-PrP antibodies 3F4 at 1:1000–1:3000 dilutions. The secondary antibody was peroxidase-conjugated rabbit anti-mouse IgG (Sigma) at a 1:80 000 dilution. Protein bands were visualized using either an enhanced chemiluminescence (ECL) or SuperSignal West Femto detection systems according to the manufactures instructions (Thermo Scientific).

## Results

### Clinical observations on CWD-inoculated transgenic mice

Clinical CWD in cervids presents primarily as a wasting syndrome with other clinical signs including changes in behavior, polyuria/polydipsia and excessive salivation [1]. In rodent prion diseases, including CWD-inoculated transgenic mice expressing deer prion protein, we typically see progressive weight loss, ataxia and gait abnormalities, limb weakness, kyphosis and somnolence. Therefore, CWD-inoculated tg66 and tgRM mice expressing human PrP were followed closely for signs of wasting, weakness, neurologic disease and behavioral changes. Fifteen tg66 mice and three tgRM mice fit our criteria as prion disease suspects (Table 1). However, this observation was complicated by the fact that aged mice often show age-related changes which are difficult to distinguish from neurologic disease [25]. Brain tissue from euthanized mice was screened for prion disease using the RT-QuIC assay for PrP amyloid seeding activity, neuropathology, and detection of disease-associated prion protein (PrPSc) by IHC and immunoblot.

### RT-QuIC analysis of tg66 and tgRM brain

The RT-QuIC assay is a recently developed, ultra-sensitive screening assay for detection of PrP amyloid seeding activity which correlates very well with presence of prion infectivity detectible by bioassay [29]. To screen for prion transmission in brain tissue from CWD-inoculated tg66 and tgRM mice, we performed RT-QuIC assays using two different recombinant PrP substrates, N-terminally truncated (90–231) hamster recombinant PrP (Ha rPrP) and full length (23–230) bank vole PrP (BV rPrP). Both Ha and BV rPrP substrates have been shown to work well with human TSE agents [27, 30] and cervid-derived CWD, including two of the CWD inocula used in the current experiments (MD-1 and WTD-1) [15].

**Figure 2:**
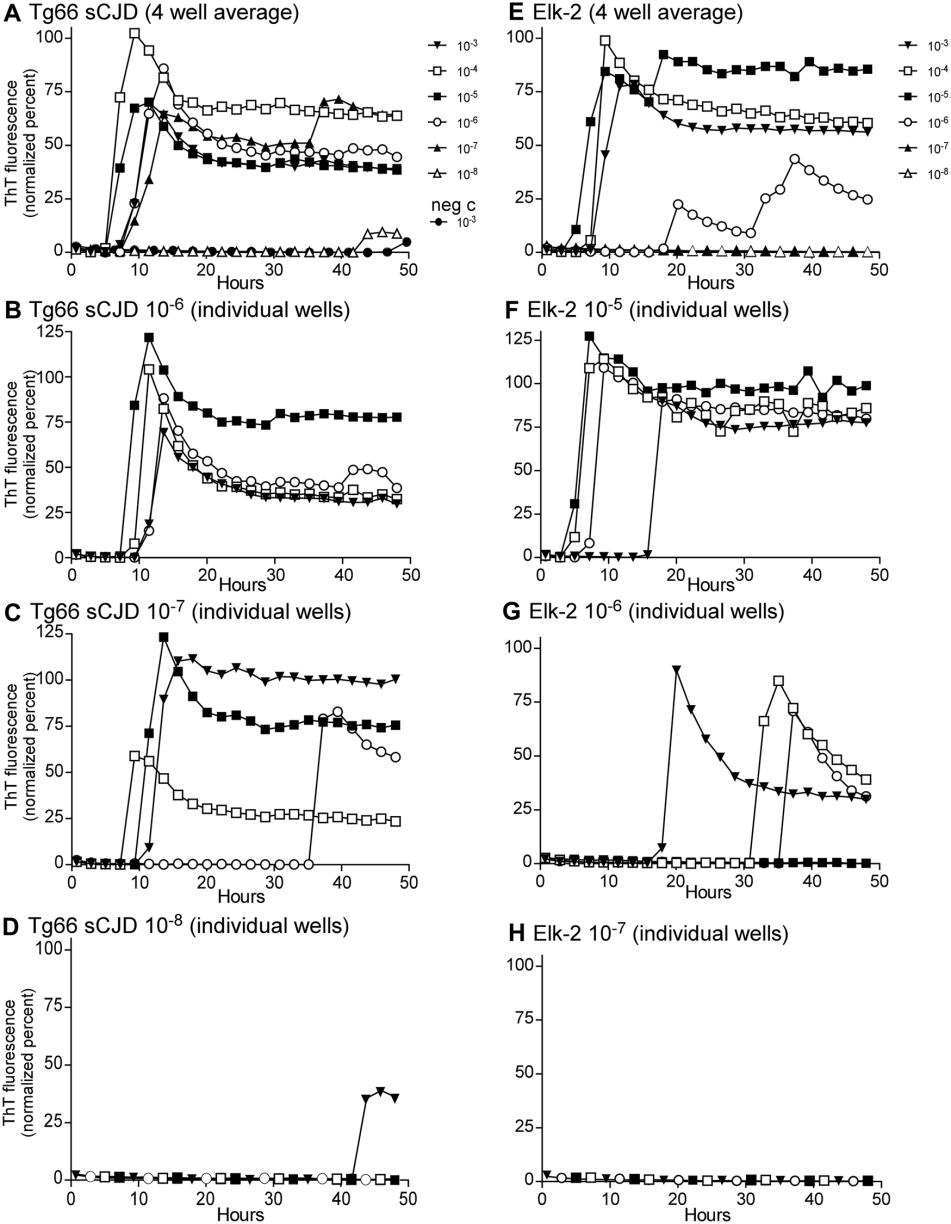
**RT-QuIC analysis for PrP amyloid seeding activity in brains from sCJD-infected tg66 mice or CWD-positive elk brain. A**–**D** The sCJD-infected tg66 brain (B946-1) and panels E-H show the CWD-positive elk brain pool (Elk-2). In **A** and **E**, a ten-fold dilution series is shown. In these panels, each curve represents and average fluorescence of four replicate wells per dilution. Dilutions are indicated to the right of each panel. **B**–**D** and **F**–**H** Detailed data from individual dilutions (indicated in the panel titles). In these panels, four wells were tested at each dilution and each curve represents data from an individual well. Note how the ratio of wells with amyloid seeding activity decreases at the sample becomes more dilute (for example, compare **F**–**H**). For all the data shown, RT-QuIC was performed using Ha rPrP 90–231 substrate.

To demonstrate the effectiveness of the RT-QuIC assay on brain tissue from transgenic mice expressing human PrP and CWD-prions from cervids, tg66 brain from clinically ill mice injected previously with human sCJD (#B946-1) and from CWD-infected elk brain (stock Elk-2) were analyzed by RT-QuIC using serial dilutions. Results using the hamster rPrP substrate are shown in Figure 2. Panels A and E show the average of four wells at each dilution tested, and results showed that these stocks reached near endpoint at dilutions of 10^−7^ and 10^−6^, respectively. In panels B–D and F–H, the curves for the individual wells for each of the stocks are shown for clarity. The titration of the MD-1 and WTD-1 CWD stocks also used as inocula in the current experiments have been previously published [15].

**Figure 3:**
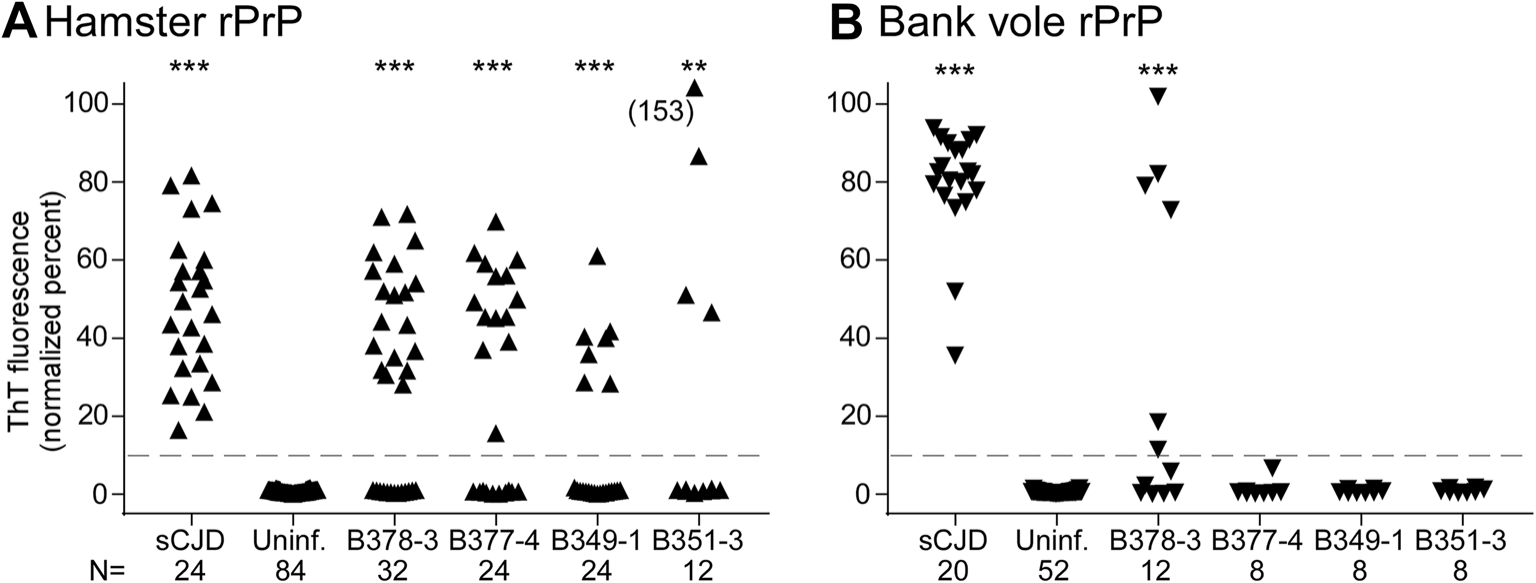
**RT-QuIC data from sCJD-infected, uninfected and indeterminate CWD-inoculated tg66 mice.** Additional RT-QuIC analysis was performed on four CWD-inoculated mice that gave positive amyloid seeding activity results on our initial screening runs to assess reproducibility of the data. Each point represents the normalized fluorescence value for an individual well measured at 25 h of RT-QuIC reaction time. Results from 3 to 4 independent assays testing 4–16 wells per mouse per assay have been pooled. The total number of wells tested for each mouse and the number of positive and negative control wells present on the same trays is provided below the x-axis. Statistical analysis was performed comparing data from individual mice to the uninfected control mice using Fisher’s exact test, *** indicates *p* < 0.0001, ** indicates *p* = 0.0013.

All CWD-inoculated transgenic mice were screened using both Ha and BV rPrP substrates. Of 88 tg66 and tgRM mice injected with CWD and tested by RT-QuIC, 75 mice had no wells with normalized fluorescence values greater than 10% for either substrate, and 13 mice had one or more wells positive with one or both substrates (Additional file 1). These 13 mice were retested, and four mice were found to have additional positive wells in this second run as well as in other additional runs using larger numbers of wells in some cases. The fraction of positive wells in each assay is shown in Additional file 1. The quantitative fluorescence value at 25 h for each well of the four mice with multiple positive wells is shown in Figure 3 and Table 2. Analysis of these data by Fisher’s exact test indicated that all four of these mice were significantly different from negative controls when tested with the hamster rPrP substrate, but only one mouse was significantly different using the bank vole rPrP substrate. Because these mice all had a mixture of positive and negative wells in these assays, they are very near the limit of detection and cannot be unequivocally determined to have replicated the injected CWD agent as there may be other explanations for these data (see “Discussion”). Therefore, these mice were considered “indeterminate” pending more experiments including second in vivo passages which will be required to further elucidate the status of CWD transmission in these mice.

### Study of neuropathology and PrP staining in brain tissue from tg66 and tgRM mice

**Figure 4:**
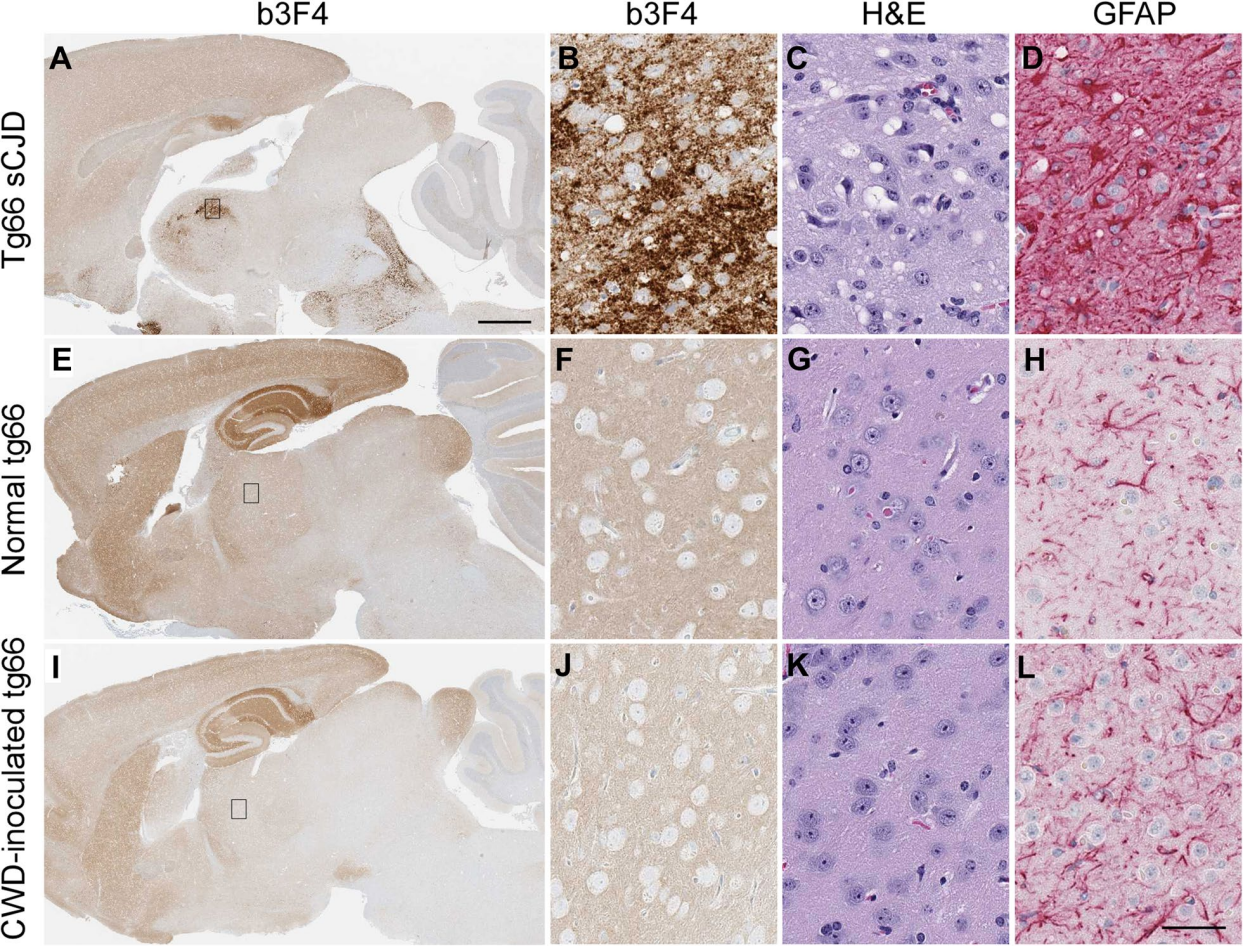
**Immunohistochemical staining and neuropathology in sCJD and CWD-inoculated tg66 mice.** Whole brain sections stained with anti-prion protein antibody b3F4 are shown in **A**, **E** and **I**. The small rectangle shown in the whole brain sections depicts the region of thalamus shown at higher magnification on the right. Thalamus panels were stained with b3F4, H&E or GFAP (shown above each column). Tissues were stained with the anti-PrP antibody to detect PrP deposition, by H&E to look for spongiosis/vacuolation and general neuropathology, and anti-GFAP was used to detect activated astrocytes. **A**–**D** Are from a tg66 mouse infected with sCJD, **E**–**H** are from a normal, aged tg66 mouse and **I**–**L** are from an aged CWD-inoculated tg66 mouse. No pathology, spongiform lesions, or excessive astroglial activation was observed in any of the uninoculated or CWD-inoculated tg66 brains. PrPsen could be seen in all brains as a smooth brown blush using anti-PrP antibody b3F4. The scale bar in **A** is 1 mm and applies to **A**, **E** and **I**. The scale bar in **L** is 50 µm and applies to **B**–**D**, **F**–**H**, and **J**–**L**.

**Figure 5:**
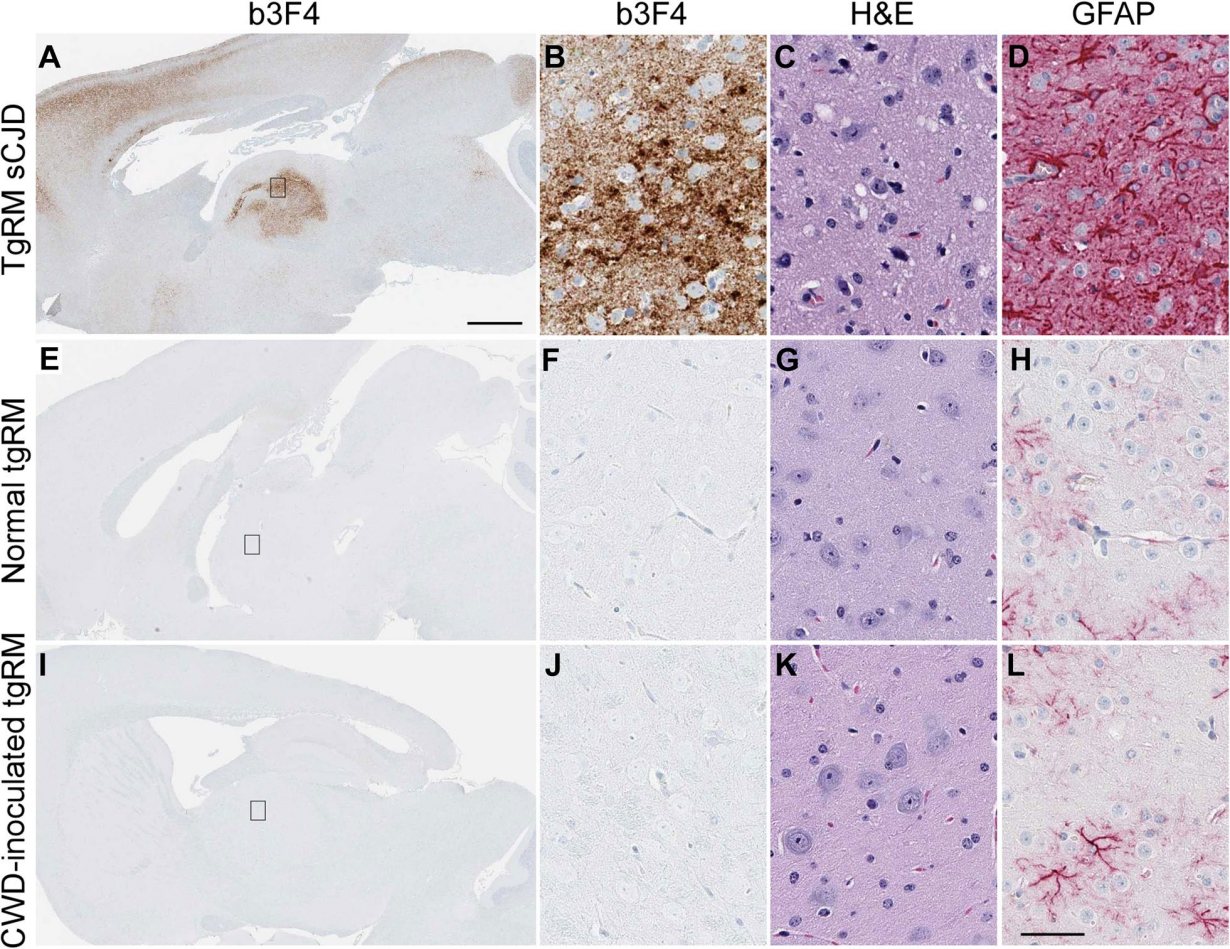
**Immunohistochemical staining and neuropathology in sCJD and CWD-inoculated tgRM mice.** Whole brain sections stained with anti-prion protein antibody b3F4 are shown in **A**, **E** and **I**. The small rectangle shown in the whole brain sections depicts the region of thalamus shown at higher magnification on the right. Thalamus panels were stained with b3F4, H&E or GFAP (shown above each column). Tissues were stained with the anti-PrP antibody to detect PrP deposition, by H&E to look for spongiosis/vacuolation and general neuropathology, and anti-GFAP was used to detect activated astrocytes. **A**–**D** Are from a tgRM mouse infected with sCJD, **E**–**H** are from a normal, aged tgRM mouse and **I**–**L** are from an aged CWD-inoculated tgRM mouse. No pathology, spongiform lesions, or excessive astroglial activation was observed in any of the uninoculated or CWD-inoculated tgRM brains. The PrPsen background staining seen in tg66 mice is not present in tgRM mice using the same staining methods. This is likely due to the lower PrPsen expression in tgRM mice. The scale bar in **A** is 1 mm and applies to **A**, **E** and **I**. The scale bar in **L** is 50 µm and applies to **B**–**D**, **F**–**H** and **J**–**L**.

In addition to RT-QuIC, we also used standard histopathology and immunoblotting to evaluate CWD infection status of humanized transgenic mice. Prion disease diagnosis is supported by the presence of various neuropathological features in brain. These include typical spongiform lesions in gray matter, astrogliosis, microgliosis and deposition of PrPSc detectable by IHC. To test for evidence of infection in transgenic mice inoculated with CWD, brains from 35 tg66 and 25 tgRM mice were analyzed for neuropathology and deposition of PrPSc. Brains were stained with two different antibodies (b3F4 and EP1802Y) to detect prion protein, GFAP for visualization of astrocytes and H&E for overall neuropathology and assessment of spongiform lesions. Brains from clinically ill, sCJD-infected tg66 (Figures 4A–D) and tgRM (Figures 5A–D) mice were used as positive controls and to demonstrate typical neuropathology found in prion-infected brains. Age-matched, uninoculated tg66 (Figures 4E–H) and tgRM (Figures 5E–H) mice were used as negative controls to determine baseline levels of normal prion protein (PrPsen) staining and identification of any non-disease-associated PrPsen aggregates. No disease-associated PrP deposition, astrogliosis or gray matter spongiform degeneration was observed in any CWD-inoculated tg66 (Figures 4I–L and Table 3) or tgRM (Figures 5I–L and Table 3) mice, including the four RT-QuIC indeterminate mice. Astroglial (GFAP) staining was greater in tg66 mice compared to tgRM mice, but no differences were noted between uninoculated controls and CWD-inoculated mice within each strain (Figures 4H, L and 5H, L). Biotinylated 3F4 staining of uninoculated tg66 and CWD-inoculated tg66 mice was similar, as all mice had a smooth distribution of PrPsen, and hippocampal and cerebral cortex stained stronger than other brain regions (Figures 4E and F compared to I, J). In addition to the smooth PrPsen staining, occasional pericellular and cytoplasmic aggregations of PrP were observed in both CWD-inoculated and uninoculated tg66 mice. Biotinylated 3F4 staining of uninoculated and CWD-inoculated tgRM mice showed little to no PrP staining (Figures 5E, F, I, J). However, tgRM mice infected with sCJD did have strong PrP staining (Figures 5A and B).

**Table 3:** **Summary of prion disease test results from tg66 and tgRM mice inoculated with CWD**

Mouse strain	Inoculum	RT-QuIC^a^	IHC^b^	WB^c^
Tg66	Elk-2	2/17	0/13	0/11
Tg66	MD-1	0/18	0/8	0/2
Tg66	WTD-1	2/15	0/14	0/3
Tg66	None	0/14	0/6	0/3
TgRM	Elk-2	0/14	0/7	0/3
TgRM	MD-1	0/13	0/9	0/2
TgRM	WTD-1	0/11	0/9	0/3
TgRM	None	0/4	0/3	0/1

^a^The number of RT-QuIC mice with results significantly different than uninfected controls is shown over the total number of mice screened from the group.

^b^The number of mice positive for neuropathology including abnormal prion protein accumulation, spongiform degeneration and astrogliosis over the number tested.

^c^The number of mice that were positive for PrPres in brain by immunoblot over the number tested.

### Analysis of protease-resistant PrP in brain by immunoblotting

**Figure 6:**
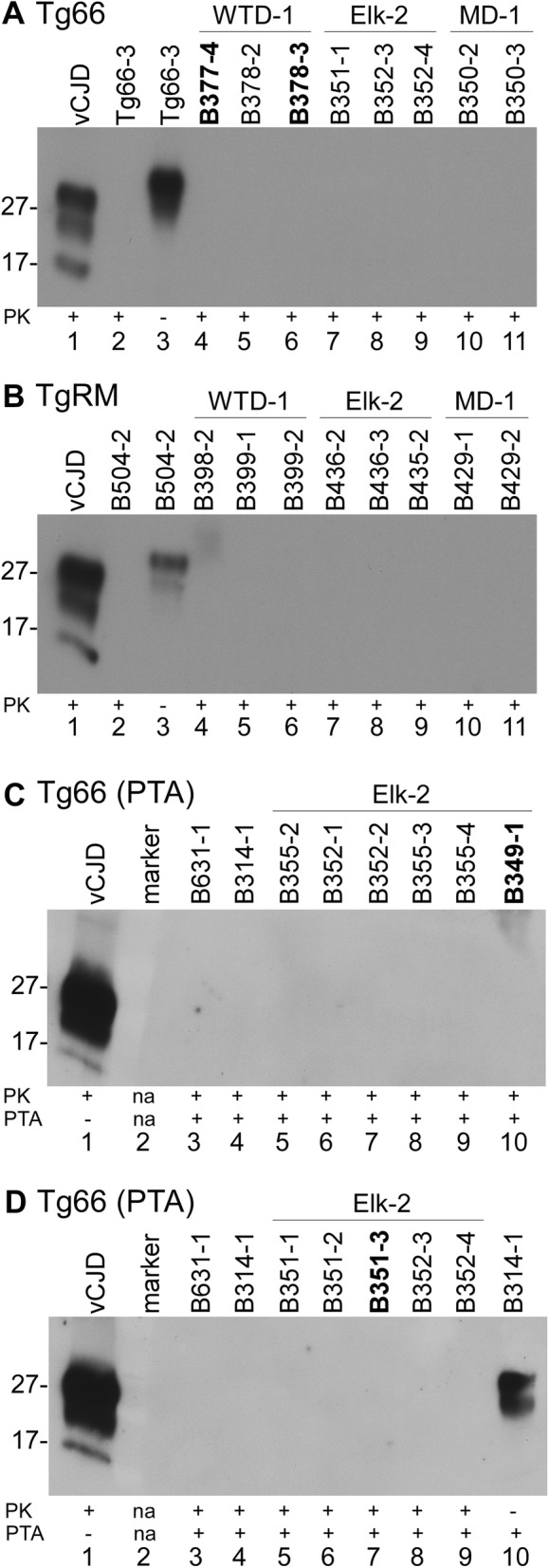
**Immunoblot screening for PrPres in CWD-inoculated tg66 and tgRM mice.** Brains from CWD-inoculated transgenic mice were treated with proteinase K (PK) or untreated and processed for immunoblot as described in the methods. **A**, **B** Results without PTA precipitation, **C**, **D** used PTA precipitation to concentrate potential low levels of PrPres. CWD inoculum and mouse numbers are shown across the top of each immunoblot. PK and PTA treatment status are shown across the bottom. **A** (tg66 mice): lane 1, tg66 mouse infected with vCJD; lanes 2, 3 uninoculated tg66 mouse; lanes 4–11 show several tg66 CWD-inoculated mice, including two RT-QuIC suspect mice (bolded). **B** (tgRM mice): lane 1, tg66 mouse infected with vCJD; lanes 2, 3 uninoculated tgRM mouse; lanes 4–11 show several tgRM CWD-inoculated mice. For both **A** and **B**, lane 1 was loaded with 0.36 mg tissue equivalents (te), lanes 2 and 4–11 were loaded with 0.72 mg te each and lane 3 was loaded with 0.04 mg te. **C**, **D** (PTA precipitation of tg66 mice): lane 1, tg66 mouse infected with vCJD; lanes 3, 4 uninoculated, age-matched tg66 mice; lanes 5–10 in **C** and 5–9 in **D** show several tg66 CWD-inoculated mice, including two RT-QuIC suspect mice (bolded). Lane 10 in **D** shows PTA precipitated PrPsen from an uninfected tg66 mouse. For **C** and **D**, lane 1 was loaded with 0.24 mg te, lanes 2–10 in **C** and lanes 2–9 in **D** were loaded with 22.7 mg te and lane 10 in **D** was loaded with 1.35 mg te. The immunoblots were probed with anti-PrP antibody 3F4 and developed using a femto detection system.

To screen the transgenic mouse brains for the presence of proteinase K-resistant PrP (PrPres), we performed immunoblots on proteinase K (PK) treated brain homogenates with or without sodium phosphotungstic acid (PTA) precipitation from a subset of both CWD-inoculated tg66 and tgRM mice (Figure 6). Two of the RT-QuIC suspect mice are shown in Figure 6A without PTA precipitation and the other two suspect mice are shown in Figures 6C and D following PK treatment and PTA precipitation. No positive immunoblot signals were observed in any of the CWD-inoculated mice screened using either immunoblot testing method (Table 3).

## Discussion

As the number of CWD-infected cervids continues to increase so does the potential for human exposure to cervid tissues containing CWD prions. Understanding the risk CWD may pose to human health is critical to avoid a repeat of the zoonotic transmission of BSE to humans recently experienced in Europe. Numerous factors, including prion protein amino acid sequence homology, prion strain properties and routes of exposures can impact whether species barriers can be overcome [31–33]. As a model for CWD transmission to humans, we inoculated two strains of transgenic mice that over-expressed human prion protein intracerebrally (ic) with three different pools of CWD-infected brain. Following very long observation periods (467–798 days), a few mice showed signs consistent with neurologic or wasting disease (Table 1). However, using traditional methods for diagnosis of prion disease (IHC and immunoblot detection of PrPres), we found no neuropathology or PrPres in any mice tested (Table 3). In contrast, using the ultra-sensitive RT-QuIC assay, we discovered 4 of 50 CWD-inoculated tg66 mice with indeterminate PrP amyloid seeding activity. The seeding activity levels in the four suspect tg66 mice were only slightly above the limit of detection of the assay and were below this threshold in many of the replicated assays (Table 2 and Figure 3). The presence of low-level PrP amyloid seeding activity in some assays suggested that prion replication may have occurred in these mice. However, all explanations including adaptive or non-adaptive sub-clinical transmission [34–36], false positive reactions or persistence of residual inoculum should also be considered.

For example, low level RT-QuIC positive reactions could be due to the persistence of residual input CWD inoculum. Intracerebral inoculations of prions into PrP null mice have been shown to have residual infectivity detected by mouse bioassay present in some animals for at least 217 days in one study [37] and for over 600 days in a second laboratory (Dr Jean Manson personal communication). Additional preliminary data in our laboratory, using RT-QuIC as a read-out, showed that input inoculum can persist and maintain prion amyloid seeding activity for at least 1 month following injection in both PrP null and tg66 mice. Based on these three experiments, it seems reasonable that input CWD inoculum might remain at adequate levels to initiate PrP amyloid seeding activity in a few mice following long incubation periods. A second explanation for the positive RT-QuIC reactions might be the potential for false positive reactions. False positive reactions may arise due to aggregations of normal PrP in over-expressing mice capable of seeding the reaction or due to spontaneous amyloid seeding of the reaction substrate. We were only able to test 14 negative control tg66 mice. Perhaps, if we tested 50 negative controls, we might find a few mice with seeding activity similar to our indeterminate mice.

Another important consideration regarding the RT-QuIC data is whether the observed RT-QuIC positivity correlates with bona fide prion infectivity. In many situations tested so far, infectivity and PrP amyloid seeding activity appear to correlate well [29, 38]. However, in another situation, samples with high levels of seeding activity were not infectious [39]. Clearly, concerns and alternative explanations exist that make conclusive interpretation of low levels of seeding activity difficult. Further research, including second passage of the indeterminate samples into both the tg66 mice and cervidized mice has been initiated in order to understand the true source and infectious nature of the measured seeding activity.

Our inefficient transmission results are similar to previous reports that also tested CWD transmission to transgenic mice that expressed human prion protein [20–24]. These previous studies found no evidence of CWD transmission based on traditional IHC and immunoblot screening techniques, identical to our results. In contrast, our results using the RT-QuIC assay identified possible evidence for subclinical transmission in a small subset of our tg66 mice. Since the RT-QuIC was unavailable at the time of the previous studies, it is unclear whether the other laboratories would also find a low incidence of RT-QuIC positive mice in their experimental mice. Another distinction between our data and previous results may be differences in the transgenic mice used. Our tg66 mice expressed very high levels of PrP (8–16× physiologic), which might increase their susceptibility to cross-species transmission of CWD. This interpretation is supported by the fact that all four RT-QuIC suspects were tg66 mice rather than tgRM mice, which express human PrP at levels fourfold lower. We have also observed a similar difference in transmission between tg66 and tgRM mice inoculated with GPI-anchorless 22L mouse scrapie [26].

Collectively, most research suggests that humans are protected against CWD infection by a strong species barrier [9]. Our experimental mouse model and intracerebral route of inoculation were selected to optimize conditions for cross-species transmission. Despite the use of this high sensitivity transmission model, we were unable to show definitive evidence for transmission. However, animal models used in the present and previous studies are not infallible at predicting cross-species transmission. Particularly in the case of CWD transmission to humans, there are many differences between humans and the various animals tested, for example, host genetic differences including human PrP allelic variations, possible CWD strain variants, and differences in routes of exposure and dosages. In agreement with our work and previous studies in humanized PrP transgenic mice, epidemiological studies have not shown evidence for CWD infection in humans, even after documented occurrence of consumption of CWD-contaminated meat by a group of individuals [40]. Nevertheless, epidemiological studies should still be continued to rule out a lower incidence of infection in certain high-risk populations.

## Additional file

**Additional file 1.** **RT-QuIC results for tg66 and tgRM mice inoculated with CWD.** Includes RT-QuIC data for each individual mouse tested (88 CWD-inoculated and several negative control mice).
