# Supplementary material for: Transmission studies of chronic wasting disease to transgenic mice overexpressing human prion protein using the RT-QuIC assay
Source: Vet Res. 2019 Jan 22;50:6. doi: 10.1186/s13567-019-0626-2 (PMC6341683; doi:10.1186/s13567-019-0626-2)
Supplement: Supplementary file 1 — Additional file 1. RT-QuIC results for tg66 and tgRM mice inoculated with CWD. Includes RT-QuIC data for each individual mouse tested (88 CWD-inoculated and several negative control mice). [file 13567_2019_626_MOESM1_ESM.doc]

**Additional file 1 RT-QuIC results for tg66 and tgRM mice inoculated with CWD**

**A. CWD-inoculated tg66 mice**

| Mouse # | Strain | Inocula^1^ | DPI | BV rPrP^2^ | Ha rPrP^2^ |
| --- | --- | --- | --- | --- | --- |
| B351-1 | Tg66 | Elk-2 | 662 | 0/4 | 0/4 |
| B351-2 | Tg66 | Elk-2 | 662 | 0/4 | 0/4 |
| B351-3 | Tg66 | Elk-2 | 662 | 0/4, 0/4 | 1/4, 1/4, 2/4 |
| B352-1 | Tg66 | Elk-2 | 607 | 0/4 | 0/4 |
| B352-2 | Tg66 | Elk-2 | 607 | 0/4 | 0/4 |
| B352-3 | Tg66 | Elk-2 | 664 | 0/4 | 0/4 |
| B352-4 | Tg66 | Elk-2 | 673 | 0/4 | 0/4, 0/4 |
| B349-1 | Tg66 | Elk-2 | 651 | 0/4, 0/4 | 2/4, 0/4, 1/4, 4/12 |
| B349-2 | Tg66 | Elk-2 | 687 | 0/4 | 1/4, 0/4 |
| B349-3 | Tg66 | Elk-2 | 687 | 0/4 | 0/4 |
| B349-4 | Tg66 | Elk-2 | 687 | 0/4 | 0/4 |
| B353-2 | Tg66 | Elk-2 | 677 | 0/4, 0/4 | 0/4 |
| B353-3 | Tg66 | Elk-2 | 719 | 0/4 | 0/4 |
| B353-4 | Tg66 | Elk-2 | 719 | 0/4 | 1/4, 0/4 |
| B355-2 | Tg66 | Elk-2 | 600 | 0/4 | 0/4 |
| B355-3 | Tg66 | Elk-2 | 635 | 0/4 | 0/4 |
| B355-4 | Tg66 | Elk-2 | 635 | 0/4 | 0/4 |

| Mouse # | Strain | Inocula | DPI | BV rPrP | Ha rPrP |
| --- | --- | --- | --- | --- | --- |
| B350-1 | Tg66 | MD-1 | 695 | 0/4, 0/4 | 0/4 |
| B350-2 | Tg66 | MD-1 | 719 | 0/4, 0/4, 0/4 | 0/4 |
| B350-3 | Tg66 | MD-1 | 719 | 0/4 | 0/4 |
| B350-4 | Tg66 | MD-1 | 719 | 0/4, 0/4 | 0/4, 0/4 |
| B350-5 | Tg66 | MD-1 | 719 | 0/4 | 0/4 |
| B347-1 | Tg66 | MD-1 | 467 | 0/4 | 0/4 |
| B347-3 | Tg66 | MD-1 | 621 | 0/4 | 0/4 |
| B347-4 | Tg66 | MD-1 | 621 | 0/4 | 0/4 |
| B347-5 | Tg66 | MD-1 | 621 | 0/4 | 0/4 |
| B348-1 | Tg66 | MD-1 | 635 | 0/4 | 0/4 |
| B348-2 | Tg66 | MD-1 | 635 | 0/4, 0/4 | 1/4, 0/4 |
| B348-3 | Tg66 | MD-1 | 635 | 0/4 | 0/4 |
| B348-4 | Tg66 | MD-1 | 635 | 0/4 | 0/4 |
| B354-1 | Tg66 | MD-1 | 474 | 0/4 | 0/4 |
| B354-2 | Tg66 | MD-1 | 635 | 0/4 | 0/4 |
| B354-3 | Tg66 | MD-1 | 635 | 0/4 | 0/4 |
| B354-4 | Tg66 | MD-1 | 635 | 0/4 | 0/4 |
| B354-5 | Tg66 | MD-1 | 635 | 1/4 | 1/4, 0/4, 0/4 |

| Mouse # | Strain | Inocula | DPI | BV rPrP | Ha rPrP |
| --- | --- | --- | --- | --- | --- |
| B377-2 | Tg66 | WTD-1 | 695 | 0/4 | 0/4 |
| B377-3 | Tg66 | WTD-1 | 710 | 0/4 | 0/4 |
| B377-4 | Tg66 | WTD-1 | 710 | 0/4, 0/4 | 3/4, 1/4, 1/4, 9/12 |
| B377-5 | Tg66 | WTD-1 | 744 | 0/4 | 0/4 |
| B378-1 | Tg66 | WTD-1 | 694 | 1/4 | 0/4 |
| B378-2 | Tg66 | WTD-1 | 717 | 0/4 | 0/4 |
| B378-3 | Tg66 | WTD-1 | 717 | 2/4, 1/4, 3/4 | 8/8, 0/4, 0/4, 11/16 |
| B378-4 | Tg66 | WTD-1 | 717 | 0/4 | 0/4 |
| B378-5 | Tg66 | WTD-1 | 503 | 0/4 | 0/4 |
| B379-3 | Tg66 | WTD-1 | 695 | 0/4 | 0/4 |
| B379-4 | Tg66 | WTD-1 | 710 | 0/4 | 0/4 |
| B379-5 | Tg66 | WTD-1 | 710 | 1/4 | 0/4 |
| B380-1 | Tg66 | WTD-1 | 626 | 0/4 | 0/4 |
| B380-2 | Tg66 | WTD-1 | 710 | 0/4 | 0/4 |
| B380-4 | Tg66 | WTD-1 | 744 | 0/4 | 0/4, 0/4 |

**B. CWD-inoculated** tgRM mice

| Mouse # | Strain | Inocula | DPI | BV rPrP | Ha rPrP |
| --- | --- | --- | --- | --- | --- |
| B433-1 | TgRM | Elk-2 | 538 | 0/4 | 0/4 |
| B433-2 | TgRM | Elk-2 | 615 | 0/4 | 0/4 |
| B433-3 | TgRM | Elk-2 | 645 | 0/4 | 0/4 |
| B434-1 | TgRM | Elk-2 | 432 | 0/4 | 0/4 |
| B435-2 | TgRM | Elk-2 | 748 | 1/4 | 0/4 |
| B435-3 | TgRM | Elk-2 | 748 | 0/4 | 0/4 |
| B435-4 | TgRM | Elk-2 | 748 | 0/4 | 0/4 |
| B436-2 | TgRM | Elk-2 | 748 | 0/4 | 0/4 |
| B436-3 | TgRM | Elk-2 | 748 | 0/4 | 0/4 |
| B436-4 | TgRM | Elk-2 | 748 | 0/4 | 0/4 |
| B437-1 | TgRM | Elk-2 | 748 | 0/4 | 0/4 |
| B437-2 | TgRM | Elk-2 | 748 | 0/4 | 0/4 |
| B437-3 | TgRM | Elk-2 | 748 | 0/4 | 0/4 |
| B437-4 | TgRM | Elk-2 | 748 | 0/4, 0/4 | 1/4, 0/4 |

| Mouse # | Strain | Inocula | DPI | BV rPrP | Ha rPrP |
| --- | --- | --- | --- | --- | --- |
| B425-1 | TgRM | MD-1 | 748 | 0/4 | 0/4 |
| B426-1 | TgRM | MD-1 | 369 | 0/4 | 0/4 |
| B426-2 | TgRM | MD-1 | 660 | 0/4 | 0/4 |
| B427-3 | TgRM | MD-1 | 582 | 0/4 | 0/4 |
| B428-1 | TgRM | MD-1 | 551 | 0/4 | 0/4 |
| B429-1 | TgRM | MD-1 | 748 | 0/4 | 0/4 |
| B429-2 | TgRM | MD-1 | 748 | 0/4 | 0/4 |
| B429-3 | TgRM | MD-1 | 748 | 0/4 | 0/4 |
| B430-3 | TgRM | MD-1 | 748 | 0/4 | 0/4 |
| B431-1 | TgRM | MD-1 | 748 | 0/4 | 0/4 |
| B431-2 | TgRM | MD-1 | 748 | 0/4 | 0/4 |
| B432-1 | TgRM | MD-1 | 748 | 0/4 | 0/4 |
| B432-2 | TgRM | MD-1 | 748 | 0/4 | 1/4 |

| Mouse # | Strain | Inocula | DPI | BV rPrP | Ha rPrP |
| --- | --- | --- | --- | --- | --- |
| B397-1 | TgRM | WTD-1 | 679 | 0/4 | 0/4 |
| B397-3 | TgRM | WTD-1 | 798 | 0/4 | 0/4 |
| B396-2 | TgRM | WTD-1 | 496 | 0/4 | 0/4 |
| B396-3 | TgRM | WTD-1 | 538 | 0/4 | 0/4 |
| B398-1 | TgRM | WTD-1 | 371 | 0/4 | 0/4 |
| B398-2 | TgRM | WTD-1 | 580 | 0/4, 0/4 | 0/4, 0/4 |
| B398-3 | TgRM | WTD-1 | 580 | 0/4 | 0/4 |
| B399-1 | TgRM | WTD-1 | 798 | 0/4 | 0/4 |
| B399-2 | TgRM | WTD-1 | 798 | 0/4 | 0/4 |
| B399-3 | TgRM | WTD-1 | 798 | 0/4 | 0/4 |
| B399-4 | TgRM | WTD-1 | 798 | 0/4 | 0/4, 0/4 |

C. Uninfected control tg66 and tgRM mice

| Mouse # | Strain | Inocula | Days old | BV rPrP | Ha rPrP |
| --- | --- | --- | --- | --- | --- |
| K745 | Tg66 | None | 587 | 0/4, 0/4, 0/4, 0/4 | 0/4 |
| A276-2 | Tg66 | None | 374 | 0/4, 0/4, 0/4 | 0/4, 0/4, 0/4 |
| Tg66-2 | Tg66 | None | 131 | 0/4 | 0/4 |
| Tg66-3 | Tg66 | None | 131 | 0/4 | 0/4, 0/4 |
| B663-3 | Tg66 | None | 635 | 0/4, 0/4, 0/4 | 0/4, 0/4 |
| B664-2 | Tg66 | None | 700 | 0/4, 0/4, 0/4, 0/4 | 0/4, 0/4, 0/4 |
| B664-3 | Tg66 | None | 700 | 0/4, 0/4 | 0/4, 0/4, 0/4, 0/4 |
| B916-2 | Tg66 | NBH | 667 | 0/4 | 0/4, 0/4 |
| B916-3 | Tg66 | NBH | 667 |  | 0/4 |
| B918 | Tg66 | NBH | 657 |  | 0/4, 0/4 |
| B918-1 | Tg66 | NBH | 667 |  | 0/4, 0/4 |
| B924-1 | Tg66 | NBH | 627 |  | 0/4 |
| M522-1 | Tg66 | None | 303 |  | 0/4 |
| M522-2 | Tg66 | None | 303 |  | 0/4 |
| G922 | TgRM | None | 611 | 0/4, 0/4 | 0/4, 0/4, 0/4 |
| B504-1 | TgRM | None | 515 | 0/4, 0/4 | 0/4 |
| B504-2 | TgRM | None | 515 | 0/4, 0/4 | 0/4, 0/4, 0/4 |
| B504-4 | TgRM | None | 634 | 0/4, 0/4, 0/4 | 0/4, 0/4 |

^1^ Elk-2, MD-1 and WTD-1 were brain homogenates from CWD-infected deer and elk. NBH was normal tg66 brain.

^2^ RT-QuIC was performed using two different recombinant PrPsen substrates, bank vole 23-231 (BV) or hamster 90-231 (Ha)

Yellow rows indicate mice that received additional testing, green rows indicate mice that were tested additional times but did not appear to be consistently positive.
